# A clinical *Pseudomonas juntendi* strain with *bla*_**IMP−1**_ carried by an integrative and conjugative element in China

**DOI:** 10.3389/fmicb.2022.929800

**Published:** 2022-07-29

**Authors:** Lin Zheng, Xinfang Zhang, Lingwei Zhu, Gejin Lu, Jiayao Guan, Mingwei Liu, Jie Jing, Shiwen Sun, Ying Wang, Yang Sun, Xue Ji, Bowen Jiang, Jun Chen, Jun Liu, Ping Chen, Xuejun Guo

**Affiliations:** ^1^School of Food and Engineering, Jilin Agricultural University, Changchun, China; ^2^Key Laboratory of Jilin Province for Zoonosis Prevention and Control, Changchun Veterinary Research Institute, Chinese Academy of Agricultural Sciences, Changchun, China; ^3^China-Japan Union Hospital, Jilin University, Changchun, China

**Keywords:** *Pseudomonas juntendi*, integrative and conjugative, carbapenem-resistant, reorganization, *bla*_*IMP*−1_

## Abstract

**Objective:**

To precisely determine the species of a carbapenem-resistant *Pseudomonas* strain 1809276 isolated from the urine of a Chinese patient and analyze its integrative and conjugative element (ICE) *1276* formation mechanism.

**Methods:**

Single-molecule real-time (SMRT) sequencing was carried out on strain 18091276 to obtain the complete chromosome and plasmid (pCN1276) sequences, and average nucleotide identity (ANI) was used for precise species identification. The ICEs in GenBank with the same integrase structure as ICE *1276* were aligned. At the same time, the transfer ability of *bla*_IMP−1_ and the antibiotic sensitivity of *Pseudomonas juntendi* 18091276 were tested.

**Results:**

This bacterium was *P. juntendi*, and its drug resistance mechanism is the capture of the *accA4'* gene cassette by the Tn*402*-like type 1 integron (*IntI1*-*bla*_IMP−1_) to form In*1886* before its capture by the ΔTn*4662a*-carrying ICE *1276*. The acquisition of *bla*_IMP−1_ confers carbapenem resistance to *P. juntendi* 18091276.

**Conclusion:**

The formation of *bla*_IMP−1_-carrying ICE *1276*, its further integration into the chromosomes, and transposition and recombination of other elements promote bacterial gene accumulation and transmission.

## Introduction

*Pseudomonas sp*. is a genus of varied and complex Gram-negative bacteria commonly isolated from soil and water, with a wide host diversity, including animals and plants. It consists of 254 species (List of Prokaryotic names with Standing in Nomenclature, LPSN; www.bacterio.net) divided into three lineages and 13 groups based on the sequences of the 16S rRNA, *gyrB, rpoB*, and *rpoD* genes (Peix et al., 2018). Among them, several species of *Pseudomonas sp*. were regarded as important opportunistic pathogens affecting humans, including *Pseudomonas aeruginosa, Pseudomonas asiatica, Pseudomonas fluorescens, Pseudomonas putida, Pseudomonas cepacia, Pseudomonas stutzeri, Pseudomonas maltophilia*, and *Pseudomonas putrefaciens* (Tohya et al., 2019). Their capabilities of acquirement of exogenous genes lead to the prone of antibiotics resistance in this genus, including carbapenem antibiotics resistance, which enhances the difficulties of treatment. *Pseudomonas sp*. can acquire carbapenem resistance genes by integrons, which further can be captured by other mobile elements (such as plasmids, transposons, etc.), and then, these genes begin to spread among other bacteria.

In this study, we analyzed a *P. juntendi* strain separated from a urine specimen of a patient in China in 2018, which was tentatively identified as *P. putida* by VITEK2 (bioMerieux), and the strain carried a carbapenem resistance gene *bla*_IMP−1_. To our knowledge, this is the first report of clinical strain of *P. juntendi* carrying *bla*_IMP−1_ in a new gene context in China.

## Materials and methods

### Bacterial isolation and identification

Strain 18091276 was isolated from a urine specimen from a patient in a tertiary hospital in northeast China in 2018 and the species of the strain was determined by the part of the sequence of the 16S rRNA gene (Edwards et al., 1989). Then, a maximum likelihood (ML) evolutionary tree was constructed using MEGA 7.0 software to evaluate the sequence similarity of the 16S rRNA genes that were aligned and listed in the BLASTN program (top 100).

The minimum inhibitory concentration (MIC) of imipenem was determined by the broth microdilution method according to the Clinical and Laboratory Standards Institute (CLSI, 2020) guidelines, and Escherichia coli ATCC 25922 was used as a control. MICs of amikacin, gentamicin, meropenem, cefazolin, ceftazidime, cefotaxime, cefepime, aztreonam, ampicillin, piperacillin, amoxicillin-clavulanate, ampicillin-sulbactam, piperacillin-tazobactam, colistin, trimethoprim-sulfamethoxazole, chloramphenicol, ciprofloxacin, levofloxacin, moxifloxacin, and tetracycline were tested by BD Phoenix-100.

### Sequencing and sequence assembly

Bacterial genomic DNA was extracted from the strain 18091276 using the UltraClean Microbial Kit (Qiagen, NW, Germany) and sequenced by a PacBio RSII sequencer (Pacific Biosciences, CA, USA). The reads were assembled *de novo* by using *SMARTdenovo* (http://github.com/ruanjue/smartdenovo).

### Sequence annotation and comparison

*Rast 2.0* (Brettin et al., 2015) and *BLASTP*/*BLASTN* (Boratyn et al., 2013) searches were used to predict open reading frames (ORFs), online databases *CARD* (https://card.mcmaster.ca/; Alcock et al., 2020), and *ResFinder 4.1* (https://cge.cbs.dtu.dk/services/ResFinder/; Bortolaia et al., 2020) to find out resistance genes, and *ISfinder* (https://www-is.biotoul.fr/; Lastest Database Update 2021-9-21; Varani et al., 2011), *TnCentral* (https://tncentral.proteininformationresource.org/), and *ICEberg 2.0* (http://db-mml.sjtu.edu.cn/ICEberg/; Liu et al., 2019) were used to find out the mobile elements. Pairwise sequence comparisons were carried out by *BLASTN*. Gene organization diagrams were drawn by *Inkscape 1.0* (http://inkscape.org/en/).

### Bacterial precise species identification and evolutionary tree construction

Bacterial precise species identification was performed using the pair-wise average nucleotide identity (ANI) analysis between strain 18091276 and the reference genome (http://www.ezbiocloud.net/tools/ani). A ≥95% ANI cutoff was used to define bacterial species (Yoon et al., 2017). *CSI Phylogeny* (https://cge.cbs.dtu.dk/services/CSIPhylogeny/; Kaas et al., 2014) calls and filters single nucleotide polymorphisms (SNPs) of strain 18091276, does site validation, and infers a phylogeny based on the concatenated alignment of the high-quality SNPs. In addition, the *MUSCLE* software program was used to align multiple single-copy core-encoded proteins identified by the core-/pan-genome analysis. The aligned sequences were then subjected to phylogenetic analysis using the *TreeBeST* (Version 1.9.2) program, a neighbor-joining tree reconstruction algorithm, and 1,000 bootstrap replicates (Nandi et al., 2010).

### Conjugation experiments

Conjugation experiments were performed as described previously (Mizuno et al., 2020). Briefly, strain 18091276 was used as a donor and sodium azide-resistant *E.coli* J53 as a recipient. Donor and recipient strains were cultured overnight at 37°C separately. Then, 3 ml of 18091276 culture was mixed up with an equal volume of *E.coli* J53 culture. The mixed cells were harvested by centrifugation for 3 min at 12,000 × *g*, washed with 3 ml of lysogeny broth (LB), and resuspended in 150 μl of LB. The mixture was spotted on a 1 cm^2^ hydrophilic nylon membrane filter (Millipore) with a 0.45-μm pore size, which was then placed on an LB agar plate and then incubated for mating at 37°C for 6 h. The cells were recovered from the filter membrane and spotted on LB agar containing 100 μg/ml sodium azide and 4 μg/ml imipenem for selecting carbapenem-resistant *E. coli* transconjugant.

### Nucleotide sequence accession numbers

The complete sequence of 18091276 has been submitted to GenBank under the accession number CP091311.

## Results and discussion

After the 18091276 strain was cultured overnight at 37°C, 2 mm round protruding colonies with smooth and regular edges, non-fusion growth, non-pyocyanin production, and the absence of metallic sheen were found on brain heart infusion agar (imipenem concentration: 4 μg/ml).

**Figure 1:**
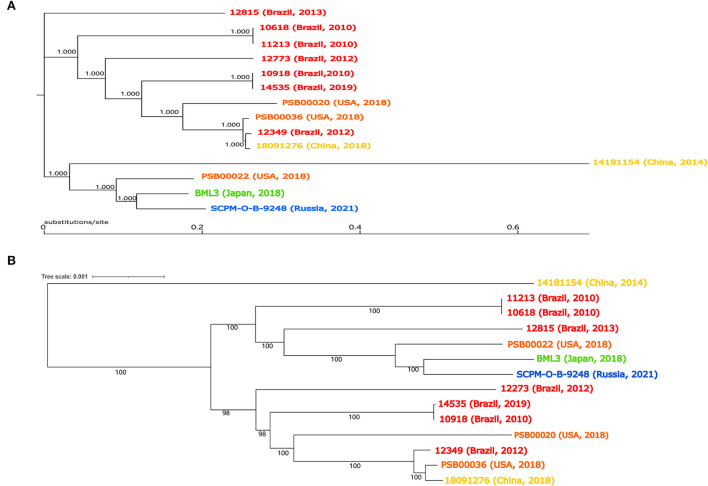
*Pseudomonas juntendi* genome phylogenetic tree. The top half is the phylogenetic tree constructed from SNPs of genomes **(A)**. The bottom half of the figure is the phylogenetic tree constructed from core genomes **(B)**. The Brazilian isolates are labeled in red, Chinese isolates are labeled in khaki, American isolates are labeled orange, Japanese isolates are labeled green, and Russian isolates are labeled blue.

Strain 18091276 was identified by the BD Phoenix-100 identification system and VITEK 2 as *P. putida*, and Table 1 shows its drug resistance spectrum. The similarity of the 16S rRNA sequence of this bacterium and *P. putida, Pseudomonas monteilii, Pseudomonas plecoglossicida*, and *P. juntendi* in the *P. putida* group was more than 99% and the strain was identified to be in the *P. putida* group (Supplementary Figure 1). After single-molecule real-time (SMRT) sequencing (basic information about SMRT sequence results was provided in Supplementary Table 1), it was found that the ANI value of strain 18091276 was more than 95% with the reference strain *P. juntendi* BML3 (GCA_009932375.1), and this strain was confirmed to be *P. juntendi* (ANI value of *P. juntendi* 18091276 were provided in Supplementary Table 2A). In 2019, Tohya M. was the first to carry out a systematic identification of *P. juntendi* and officially named this species (Tohya et al., 2019). The analysis of all 14 strains that were annotated as *P. juntendi* in GenBank (cutoff date was November 2021; Table 2) found that the clinical isolates before 2018 were from Brazil, and the isolates in China, the USA, Japan, and Russia only appeared after 2018. Although *P. juntendi* 18091276 and 14181154 were all isolated in China, they had a far phylogenetic relationship (Figure 1). According to the SNPs and core-genome phylogenetic tree, *P. juntendi* 18091276 was the closest relative to *P. juntendi* 12349 (Brazil, 2012) and *P. juntendi* PSB00036 (the USA, 2018; Figure 1). We speculate that *P. juntendi* 18091276 might be transferred from the USA or Brazil through international food (animal- and plant-based) trade and travel. The identification method at the time was not precise, many *P. juntendi* isolates were wrongly identified as *P. putida* or other species in the *P. putida* group (Morimoto et al., 2020), resulting in fewer epidemiological data and whole-genome data of this strain in other countries. ResFinder screening found that, out of the 14 *P. juntendi* strains, six Brazilian strains carried the carbapenem resistance genes *bla*_IMP_ and *bla*_VIM_ and two Chinese strains carried *bla*_IMP_ (see Table 2 for strain information), showing that *P. juntendi* could acquire *bla*_IMP_ and *bla*_VIM_, like *P. aeruginosa* and *P. putida*, and develop carbapenem resistance, and it is potentially harmful.

**Table 1:** Antimicrobial susceptibility of 18091276.

**Antimicrobial type**	**Antimicrobial**	**MIC (**μ**g/mL)**^a^	**SIR** ^b^
Aminoglycosides	Amikacin	≤ 8	S
	Gentamicin	4	S
β-lactam	Imipenem	32	R
	Meropenem	>8	R
	Cefazolin	>16	R
	Ceftazidime	>16	R
	Cefotaxime	>32	R
	Cefepime	>16	R
	Aztreonam	16	I
	Ampicillin	>16	R
	Piperacillin	8	S
	Amoxicillin-Clavulanate	>16/8	R
	Ampicillin-Sulbactam	>16/8	R
	Piperacillin-Tazobactam	8/4	S
Colistin	Colistin	1	NA
Sulfonamide	Trimethoprim-Sulfamethoxazole	>2/38	R
Chloramphenicol	Chloramphenicol	>16	R
Quinolones	Ciprofloxacin	≤ 0.5	S
	Levofloxacin	≤ 1	S
	Moxifloxacin	4	NA
Tetracycline	Tetracycline	4	S

^a^MIC, minimum inhibitory concentration.

^b^SIR, Susceptible (S), Intermediate (I), Resistant (R).

NA, not applicable.

**Table 2:** The information of *P. juntendi*.

**Strain**	**Source**	**Time**	**Country**	**Size (Mb)**	**GC content (%)**	**Resistance gene**	**Assembly ID**
10918	Urine	2010	Brazil	6.09	62	$aadA1,aph\left(3^{\prime }\right)-VIa,aacA4^{\prime },sul1,bla_{\text{IMP}-16}$, *bla*_VIM−2_*, blaOXA-129*	GCA_014062235.1
10618	Blood	2010	Brazil	5.71	62.2	*bla*_VIM−2_ *, aacA4', blaOXA-129, sul1*	GCA_014062305.1
11213	Blood	2010	Brazil	5.70	62.2	$aacA4^{\prime },sul1,bla_{\text{OXA}-129}$, *bla*_VIM−2_	GCA_014062275.1
12273	Urine	2012	Brazil	5.59	62.5		GCA_014062265.1
12349	Blood	2012	Brazil	5.64	62.3	$aacA4^{\prime },sul1,bla_{\text{IMP}-16}$, *bla*_VIM−2_	GCA_014062185.1
12815	Urine	2013	Brazil	5.69	62.5	$sul1,aadA1,aacA4^{\prime },aph\left(3^{\prime }\right)-VIa,aadA1,bla_{\text{IMP}-1}$	GCA_014062135.1
14181154	Catheter	2014	China	5.61	62.3	$aacA4^{\prime },aadA1,bla_{\text{OXA}-21}$, *bla*_IMP−1_	CP045554.1
18091276	Urine	2018	China	5.89	62.3	$aacA4^{\prime },bla_{\text{IMP}-1}$	This study
BML3	Sputum	2018	Japan	5.73	62.7		GCA_009932375.1
PSB00022	Urine	2018	USA	5.77	62.1		GCA_016009075.1
PSB00036	Urine	2018	USA	5.42	62.4		GCA_016337345.1
PSB00020	Sputum	2018	USA	6.20	62.2		GCA_016009085.1
14535	Blood	2019	Brazil	6.07	62.1	$aacA4^{\prime },sul1,bla_{\text{VIM}-2}$, *bla*_IMP−16_	GCA_014062085.1
SCPM-O-B-9248	Sputum	2021	Russia	5.67	62.3		GCA_018138545.1

**Figure 2:**
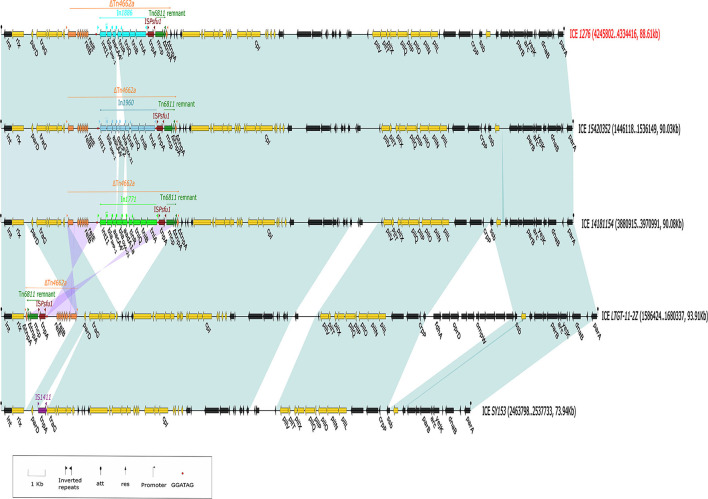
Linear alignment map of integrative and conjugative elements (ICEs). The integration region is yellow, the backbone region is black, In*1886* is light blue, In*1960* is blue, In*1771* is green, Tn*4662a* is orange, IS*Psfu1* is dark red, Tn*6811* is dark green, and IS*1411* is purple. The solid red dot is the integron inverted repeat (IR) recognition sequence GGATAG, and the shaded region represents regions with >90% nucleotide identity. Except for *Pseudomonas putida* SY153 ICE *SY153*, the other ICEs were all Tn*4662a*-derived structures. IS*1411* (ISL3 family) recognizes the 8 bp binding site (ATCAAAGG) on *traG* through IR, thereby inserting it into ICE *SY153* and causing the binding site to form directed repeat (DR). Except for the Tn*4662a*-derived structure in the ICE of *Pseudomonas* sp. LTGT-11-2Z that was inserted in the 257 bp site downstream of *rlx* after inversion, Tn*4662a*-derived structures from other strains were inserted in the positive direction 296 bp upstream of the integrating conjugative element protein of the TIGR03757 family in these ICEs. The integron IR recognition site (GATAGG) was similarly present at 1,153 bp upstream of the res site on Tn*4662a* (ICE *LTGT-11-2Z*), but no integron was inserted. A 14.29-kb accessory module (dark pink) was also inserted 74 bp upstream of the *ssb* (single-strand DNA binding protein) in the backbone region of ICE *LTGT-11-2Z* and contains *fdhA* (formate dehydrogenase), *oprD* (outer membrane porin protein), and *ompW* (outer membrane protein). Alignment of ICE *1276* with ICE *14181154* from *P. juntendi* 14181154 and ICE *15420352* from *P. putida* 15420352 found that only the number of gene cassettes captured by the Tn*402*-like integron was different, and other structures were identical.

The chromosome of *P. juntendi* 18091276 contained an 88.61 kb integrative and conjugative element (ICE) that was named ICE *1276*. ICE *1276* contained *attL* and *attR* produced from recombination, a complete integrase gene (*int)*, the F-type IV secretion system responsible for the ICE transfer, the conjugation gene *cpl*, and *rlx* responsible for autonomous rolling circle replication (Johnson and Grossman, 2015). ICE *1276* did not contain the cleavage enzyme *xis* or the origin of replication *oriT*. *crpP* (quinolone resistance gene) was also found in the backbone region of ICE *1276*. *crpP* was mainly present in the ICEs of *Pseudomonas aeruginosa* and could increase ciprofloxacin resistance (Zhu et al., 2021). However, *P. juntendi* 18091276 did not possess quinolone resistance. In addition, a 15.96-kb accessory module was discovered in ICE *1276*, and this structure had the closest phylogenetic relationship with Tn*4662a* (a Tn*3* family transposon) in plasmid pDK1 (NC_014124.1; coverage: 58%, identity: 98.65%). Moreover, it had the same 38 bp inverted repeat sequence (IRs) as Tn*4662a*, and a 5-bp (AGTAT) directed repeat sequence (DRs) was generated during insertion (detailed information is shown in Supplementary Table 3). Tn*4662a* was first found on a plasmid pDK1 carried by *P. putida* HS (Yano et al., 2010). In contrast to Tn*4662a* (pDK1), the insertion structure of *P. juntendi* 18091276 also contained a Tn*6811* remnant (Tn*3* family), a single copy insertion element IS*Psfu1* (IS*5* family), and a complete In*1886* (Tn*402*-like type 1 integron) carrying *bla*_IMP−1_ and *aacA4'*. However, the res_site_II, res_site_III, *tnpR*, and *tnpA* sequences in Tn*4662a* (ICE *1276*) were partially deleted, which should have a Tn*4662a*-derived structure (Figure 2).

Until November 2021, only 4 ICEs with the same integrase as ICE *1276* were indexed in GenBank (Table 3 shows the strain information). Interestingly, these ICE *1276*-carrying strains were *Pseudomonas* sp. from China. ANI calculator was used to precisely analyze the identified *Pseudomonas* sp. 14181154 and LTGT-11-2Z. The ANI value of *Pseudomonas sp*. 14181154 and the reference strain *P. juntendi* BML3 was more than 95% (ANI value in Supplementary Table 2B), so *Pseudomonas sp*. 14181154 was identified as *P. juntendi* (Figure 1 shows the phylogenetic relationship with other *P. juntendi* strains), but the ANI of LTGT-11-2Z and all *Pseudomonas* species were lower than 95%. Therefore, LTGT-11-2Z was only identified as *Pseudomonas sp*. All ICEs were obtained from *Pseudomonas* sp. chromosomes, and *E. coli* transconjugants were not obtained after repeated conjugation experiments. Except for the exogenous insertion sites, the remaining structure of the ICEs was nearly identical. Another exception is that a 14.29 kb accessory module was inserted at 74 bp upstream of *ssb* (single-strand DNA binding protein) in the backbone region of ICE *LTGT-11-2Z* (plant-derived *Pseudomonas sp*. LTGT-11-2Z). This ICE seemed to have caused the transfer of large fragments between *Pseudomonas* species under specific conditions.

**Table 3:** The information of strains carrying ICEs.

**Strain**	**Source**	**Species identification**		**Year**	**Country**	**Size (Mb)**	**GC content (%)**	**Assembly ID**
		**GenBank**	**This study**					
LTGT-11-2Z	Alhagi sparsifolia Shap.	*Pseudomonas sp*.	*Pseudomonas sp*.	2014	Xinjiang, China	6.07	61.7	CP033104.1
14181154	Homo sapiens, Catheter	*Pseudomonas sp*.	*P.juntendi*	2014	Hunan, China	5.61	62.3	CP045554.1
SY153	Homo sapiens, urine	*P. putida*	*P. putida*	2012	Sanya, China	5.60	62.0	CP062218.1
15420352	Homo sapiens, urine	*P. putida*	*P. putida*	2015	Hunan, China	6.17	61.6	CP045551.1
18091276	Homo sapiens, urine	*P.juntendi*	*P.juntendi*	2018	Changchun, China	5.89	62.3	This study

**Figure 3:**
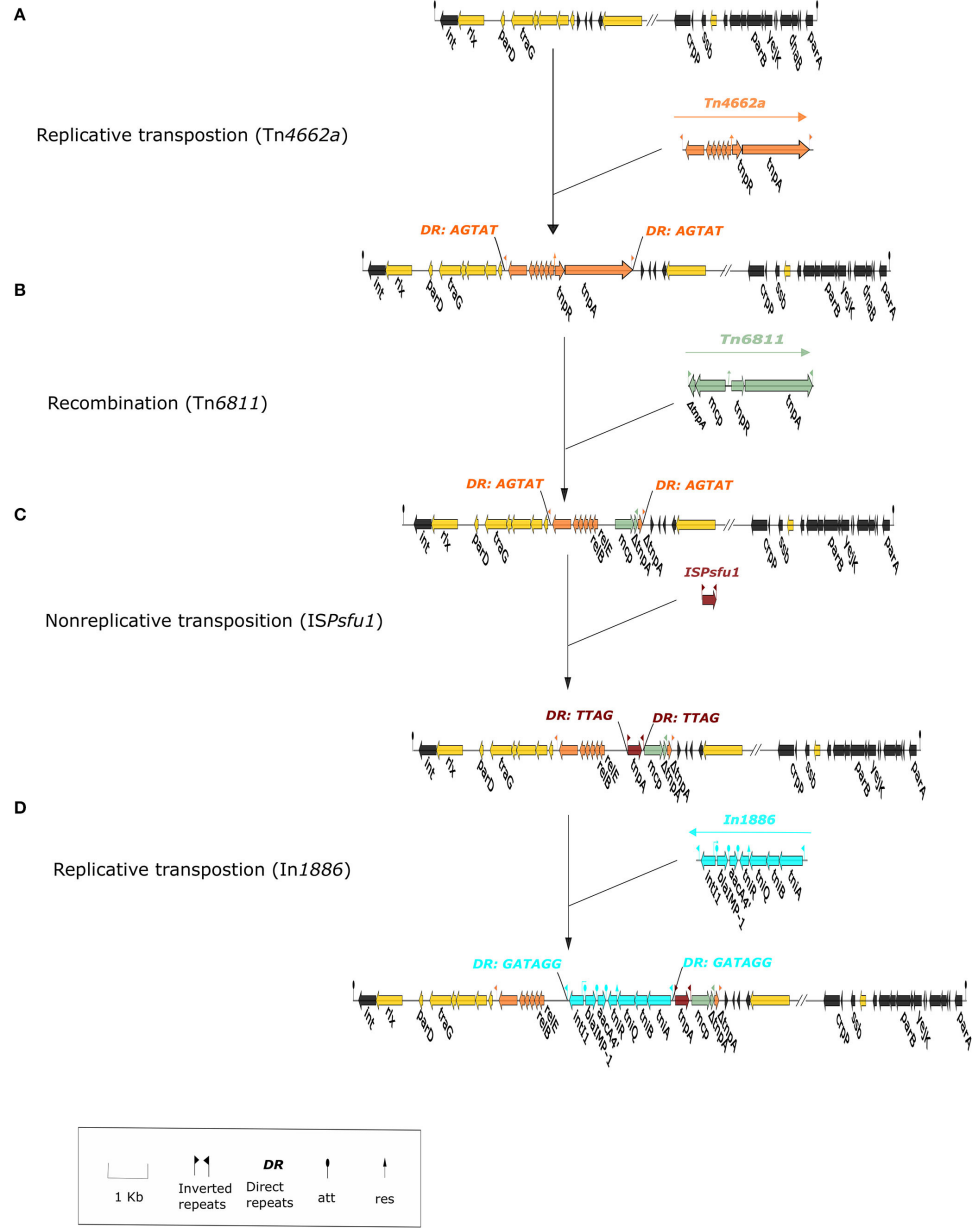
The formation process of integrative and conjugative element (ICE) *1276*. The figure shows the ICE *1276* structure as a local structure. The integration region of ICE *1276* is yellow, the backbone region is black, In*1886* is light blue; **(A)** Tn*4662a* wasrecombined in ICE *1276* through replicative transposition and produces bilateral directed repeat sequence (DRs) (AGTAT); **(B)** Tn*6811* and Tn*4662a* underwent recombination to form the ΔTn*4662a*-ΔTn*6811* structure; the truncated part of Tn*4662a* includes *tnpA*, complete deletion of *tnpR*, and the recombination sites res_site_II and res_site_III; **(C)** IS*Psfu1* was inserted into ΔTn*4662a*-ΔTn*6811* through non-replicative transposition and produced bilateral DRs (TTAG); **(D)** In*1886* was inserted upstream of the res_site_I in ΔTn*4662a* through replicative transposition and produced bilateral DRs (GATAGG).

As the downstream region of *Tn4662a* is truncated by *Tn6811*, it is unable to carry out normal replicative transposition (Grindley and Reed, 1985), but it still contains an intact res_site_I (Supplementary Table 4A) and can recombine with In*1886* and IS*Psfu1* (Grindley and Reed, 1985; Brovedan et al., 2021) (Figure 2). IRs and DRs were absent at both ends of IS*Psfu1*-In*1886* and did not constitute a composite transposon (Zong, 2014). Alignment with 10 Tn*402*-like integrons containing *bla*_IMP−1_ and *aacA4'* gene cassettes that were indexed before November 2021 (Table 4 shows the strain information) found that except for In*1960*, In*1771*, and In*1886* located in ICEs, the IS*Psfu1* element was not inserted upstream or downstream of the remaining integrons (regardless of whether it is on the plasmid or chromosome) and there were two independent insertion processes. The Tn*4662a*-drived structure in ICE *LTGT-11-2Z* (Figure 2) indicated that, after IS*Psfu1* had undergone non-replicative transposition, In*1886* was inserted upstream of the res_site_I of ΔTn*4662a* (Figure 3 shows the formation process of ICE *1276*).

**Table 4:** Genetic contexts of In*1886*.

**Strain**	**Country**	**Year**	**Source**	**Location**	**Structure**	**Genbank AN**
*P. aeruginosa* PA15W	China	NA	Human	P	*Int1*-*bla*_IMP−1_-*aacA4*'-*Tni402*	MN961673
P. juntendi 18091276	China	2018	Urine	C	*Int1*-*bla*_IMP−1_-*aacA4*'-*Tni402*-IS*Psful*	In this study
*Pseudomonas* sp. 14181154	China	2014	Catheter	C	*Int1*-*bla*_IMP−1_-*aacA4'*-*bla*_OXA−21_-*aadA1a*-*Tni402*-IS*Psful*	CP045554.1
*P. putida* 15420352	China	2015	Urine	C	*Int1*-*bla*_IMP−1_-*aacA4'*-*qacF2*-*bla*_OXA−21_-*Tni402*-IS*Psful*	CP045551.1
*K. pneumoniae* 2013050801	China	2013	Blood	P	*Intl1*-*bla*_IMP−1_-*guc162*-*aacA4'*-*aadA6*-*Tni402*	KT345947
*K. oxytoca* 7121	China	2014	Sputum	P	*Intl1*-*bla*_IMP−1_-*guc162*-*aacA4'*-*aadA6*-*Tni402*	KX784502
*P. aeruginosa* 97	Ghana, Western Africa	2015	Urine	C	*Intl1*-*bla*_OXA−10_/*aacA4*-*bla*_IMP−1_-*Tni402*	CP031449
*P. aeruginosa* JUNP133	Japan	NA	NA	C	*Intl1*-*bla*_OXA−10_/*aacA4*-*bla*_IMP−1_-*Tni402*	LC636409
*E. coli* MBL1-07^a^	India	2008	NA	NA	*Intl1*-*bla*_OXA−10_/*aacA4*-*bla*_IMP−1_	LC169568
*P. alcaligenes* KAM426	Japan	2020	wastewater	C	*Int1*-*fosE*-*aacA4'-3-aacA4'-3*-*blaIMP-1*-*qacG8*-*qacE*Δ*1-sul1-Tni402*	AP024354

^a^Since the uploaded of this strain was only an integrator sequence, unable to determine the mobile genetic elements upstream and downstream.

P, Plasmid.

C, Chromosome.

NA, not applicable.

**Figure 4:**
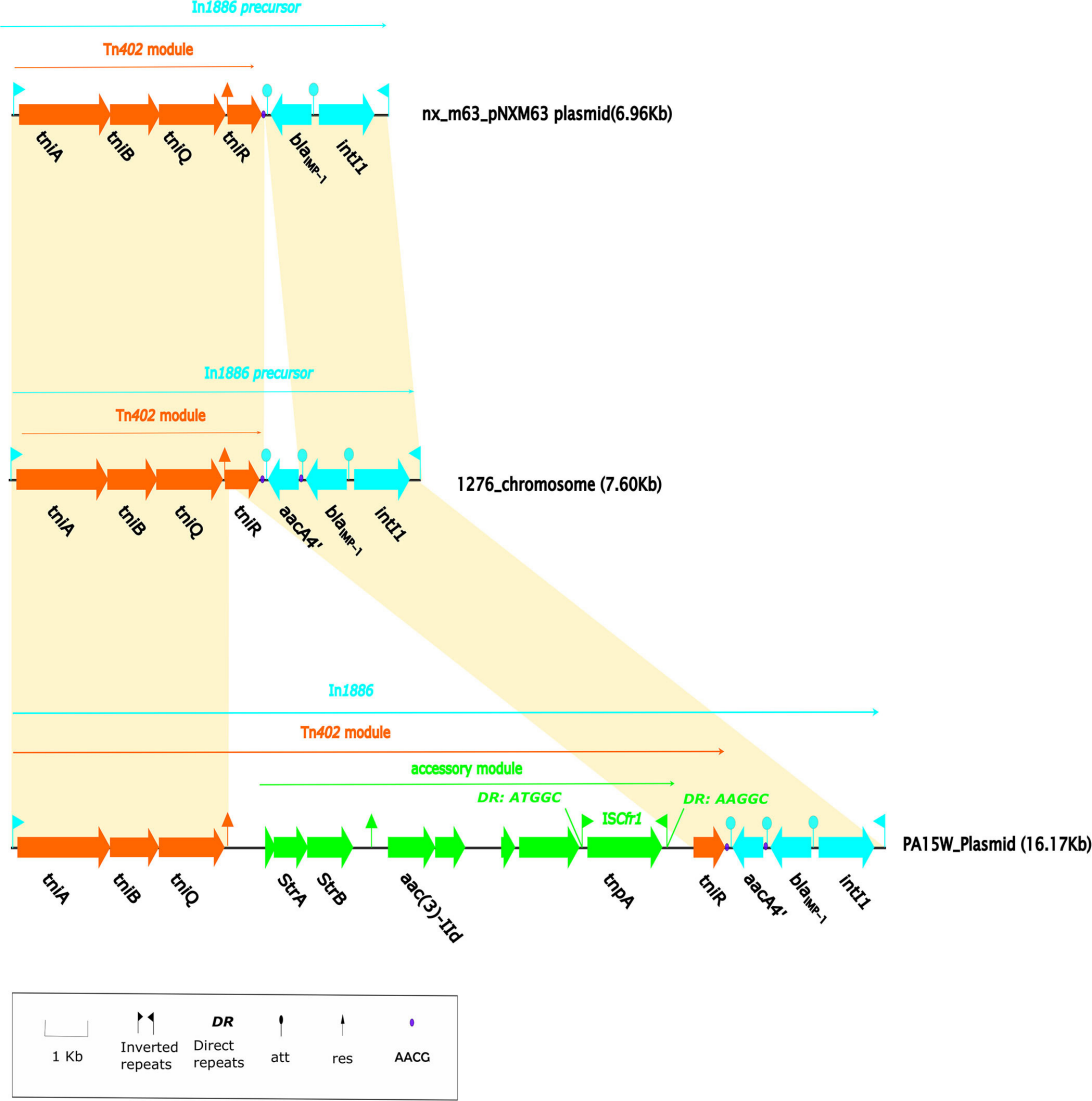
Linear alignment map of In*1886*. The Tn*402* transposition module is orange-red, the 3'-CS of the integron is blue, and the exogenous insertion element is green. The solid purple dot is the AACG sequence that is recognized by the gene cassette *att* and forms two copies in *In1886* due to the insertion of the *aacA4'* gene cassette. The shaded region represents a homologous region (>90% nucleotide identity). The pNXM63 (MW150990) from *Morganella morganii* nx_m63 contains an *intI1*-*bla*_IMP−1_-*tniABQR* structure. When this integron was compared with In*1886*, except for the gene cassette *aacA4'*, all of the bases were completely identical, and there was no missing base. At the same time, the downstream region of the gene cassette *bla*_IMP−1_ in this integron contained the AACG site, which was present on both sides of the gene cassette *aacA4'* in In*1886*. In addition, In*1886* was also present in the plasmid (GenBank accession no.: MN961673) in *P. aeruginosa* PA15W. However, a 7.45-kb exogenous insertion structure was inserted in the res_site_r1 recombination site of the Tn*402* mobile module in the Tn*402* of PA15W-In*1886*, causing res_site_r1 to be truncated (Supplementary Table 2). This exogenous insertion structure contained eight open reading frames (ORFs), including a complete IS*Cfr1* (IS1182 family), aminoglycoside resistance genes *strA* and *strB*, and *aac(3)-IId*.

IMP-1 was first discovered in a *P. aeruginosa* strain from Japan (Walsh et al., 2005) and is now the most widely circulating IMP enzyme in Southeast Asia (Walsh et al., 2005). Up until November 2021, 86 IMP family members were indexed in GenBank, and most *bla*_IMP−1_ were carried and transferred by type 1 integrons (Diene and Rolain, 2014). After the Tn*402* integron captures *bla*_IMP−1_, Tn*402* possesses the self-transfer capability and can be integrated into transposons or res sites on plasmids to expand its transmission range (Gillings, 2017). In 2014, pNXM63 (MW150990) from *Morganella morganii* nx_m63 that was isolated in a hospital in China contained a Tn402-like type 1 integron (intI1-*bla*_IMP−1_; Xiang et al., 2021). Based on the repeat sequence (AACG) on both sides of the gene cassette, it can be deduced that In*1886* is a structure formed after the integron on pNXM63 captured the *aacA4'* gene cassette (Figure 4). The Tn*402*-like transposon module at the 3' CS end of In*1886* could capture other drug resistance genes. An example is In*1886* in the PA15W plasmid in *P. aeruginosa* (GeneBank accession no.: MN961673). However, as this In*1886* IS*Cfr1* fragment mediated the insertion of *strA*-*strB*-*aac(3)-IId* to the Tn*402* recombination site on PA15W-In*1886*, it resulted in the truncation of the res_site_r1 site (Figure 4, Supplementary Table 4B), leading to irreversible recombination (Rowland et al., 2020). In*1886* can also capture *aadA6, bla*_*OXA*−21_, *aadA1a, qacF2*, and *guc162* gene cassettes through recombination at specific sites to obtain a series of derived structures (Labbate et al., 2009; Table 4). This integron and its derived structures were mostly present on *Pseudomonas sp*. chromosomes and were also discovered in *K. pneumoniae* and *K. oxytoca* plasmids (Table 4).

Therefore, *bla*_IMP−1_ can be recombined upstream of res_site_I in Tn*4662a* using a Tn*402*-like integron as a medium to achieve transfer from other species to *P. juntendi* 18091276. After that, large fragments are transferred between *Pseudomonas* sp. through ICEs. The Tn*4662a*-derived structures in these ICEs contain the intact res_site_I sequence. It can also continue recombination with other elements. In addition, it was also found that, in addition to capturing gene cassettes, the res_site_r1 on *tniABQR* at 3' CS can also undergo further recombination, resulting in the production of multidrug resistance. Regarding different hosts, ICE *1276*-like integration elements can also undergo recombination with exogenous genes through *ssb* to carry out adaptive evolution.

## Conclusion

This study was the first to report a clinical isolate of *P. juntendi* in China. At the same time, it was found that this bacterium can capture Tn*402*-like type 1 integron containing *bla*_*IMP*−1_ through the ICE of Tn*4662a*. This provides a new vector and host for the horizontal transfer of *bla*_IMP−1_. Hence, there is a need to improve bacterial identification methods and drug resistance monitoring in opportunistic pathogens in hospitals. At the same time, the ICE *1276*-Tn*4662a*-In*1886* structure and its mutant-derived structures should be closely monitored, particularly its epidemiology in China.

## Data availability statement

The datasets presented in this study can be found in online repositories. The names of the repository/repositories and accession number(s) can be found at: https://www.ncbi.nlm.nih.gov/genbank/, CP091311.

## Author contributions

All strains were provided by China-Japan Union Hospital, Jilin University. PC, XG, JC, JL, and LZhu conceived, directed, and carried out the study. ML, GL, JJ, YW, XZ, and BJ prepared samples for sequence analysis. JG, SS, and LZhe acquired samples and analyzed the data. All authors have read and approved the final manuscript.

## Funding

Funding for study design, data collection, data generation, and publication costs was provided by the National Science and Natural Science Foundation of China (Grant agreement 31872486).

## Conflict of interest

The authors declare that the research was conducted in the absence of any commercial or financial relationships that could be construed as a potential conflict of interest.

## Publisher's note

All claims expressed in this article are solely those of the authors and do not necessarily represent those of their affiliated organizations, or those of the publisher, the editors and the reviewers. Any product that may be evaluated in this article, or claim that may be made by its manufacturer, is not guaranteed or endorsed by the publisher.
