# Supplementary material for: A clinical Pseudomonas juntendi strain with blaIMP−1 carried by an integrative and conjugative element in China
Source: Front Microbiol. 2022 Jul 29;13:929800. doi: 10.3389/fmicb.2022.929800 (PMC9374279; doi:10.3389/fmicb.2022.929800)
Supplement: Supplementary file 1 [file Table_1.DOCX]

**
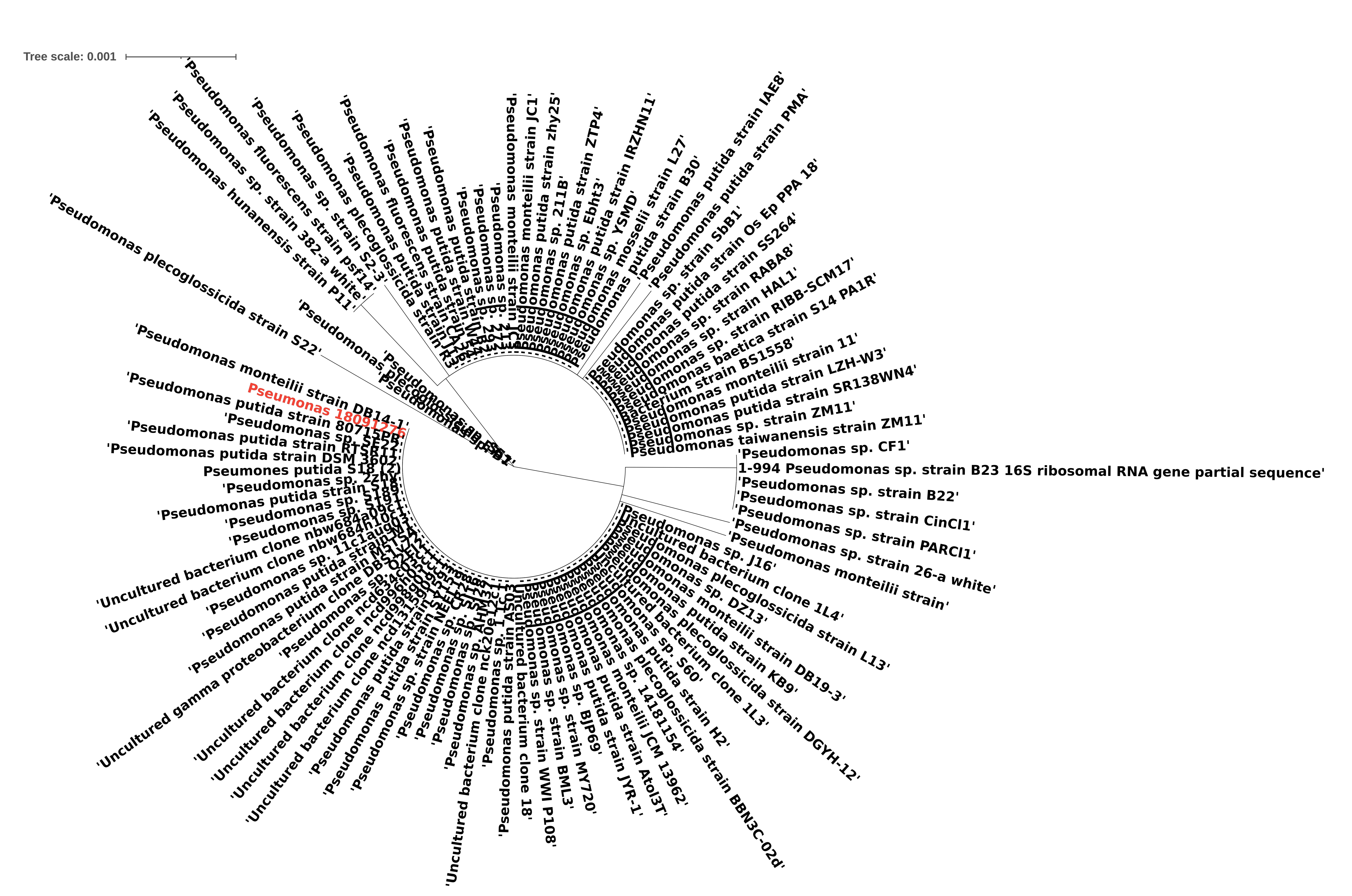
**

**Supplement Figure S1** **16S rRNA phylogenetic tree of *Pseudomonas putida* group.** Strain 18091276 is labeled red in the phylogenetic tree.
